# Innovative Stacked Yellow and Blue Mini-LED Chip for White Lamp Applications

**DOI:** 10.3390/mi15060796

**Published:** 2024-06-17

**Authors:** Tzu-Yi Lee, Chien-Chi Huang, Wen-Chien Miao, Fu-He Hsiao, Chia-Hung Tsai, Yu-Ying Hung, Fang-Chung Chen, Chun-Liang Lin, Kazuhiro Ohkawa, Jr-Hau He, Yu-Heng Hong, Hao-Chung Kuo

**Affiliations:** 1 Department of Photonics, College of Electrical and Computer Engineering, National Yang Ming Chiao Tung University, Hsinchu 30010, Taiwan; alex860512.ee10@nycu.edu.tw (T.-Y.L.); chienchi28@gmail.com (C.-C.H.); s.tsai@smartkem.com (C.-H.T.); white.white.ee11@nycu.edu.tw (Y.-Y.H.); fcchendop@nycu.edu.tw (F.-C.C.); 2 Semiconductor Research Center, Foxconn Research, Taipei 11492, Taiwan; leona.wc.miao@foxconn.com (W.-C.M.); harry.fh.hsiao@foxconn.com (F.-H.H.); 3 Department of Electrophysics, College of Science, National Yang Ming Chiao Tung University, Hsinchu 30010, Taiwan; clin@nycu.edu.tw; 4 Computer, Electrical and Mathematical Sciences and Engineering (CEMSE) Division, King Abdullah University of Science and Technology (KAUST), Thuwal 23955-6900, Saudi Arabia; kazuhiro.ohkawa@kaust.edu.sa; 5 Department of Materials Science and Engineering, City University of Hong Kong, Tat Chee Avenue, Kowloon, Hong Kong, China; jrhauhe@cityu.edu.hk

**Keywords:** mini-LED, vertical stacked, InGaN yellow LED, reliability

## Abstract

This study introduces a novel approach for fabricating vertically stacked mini-LED arrays, integrating InGaN yellow and blue epitaxial layers with a stress buffer layer to enhance optoelectronic characteristics and structural stability. This method significantly simplifies the LED design by reducing the need for RGB configurations, thus lowering costs and system complexity. Employing vertical stacking integration technology, the design achieves high-density, efficient white light production suitable for multifunctional applications, including automotive lighting and outdoor signage. Experimental results demonstrate the exceptional performance of the stacked yellow and blue mini-LEDs in terms of luminous efficiency, wavelength precision, and thermal stability. The study also explores the performance of these LEDs under varying temperature conditions and their long-term reliability, indicating that InGaN-based yellow LEDs offer superior performance over traditional AlGaInP yellow LEDs, particularly in high-temperature environments. This technology promises significant advancements in the design and application of lighting systems, with potential implications for both automotive and general illumination markets.

## 1. Introduction

In recent years, advancements in technology have fueled increasing demand for high-performance lighting systems in the automotive industry. Miniature light-emitting diodes (mini-LEDs) are emerging as a promising solution for automotive lighting due to their high brightness, rapid response time, low energy consumption, and broad color gamut [1]. This technology is not only suitable for in-vehicle displays, but also extends to outdoor billboards and lighting fixtures [2,3]. Typically, white light is produced using GaN blue LED wafers coated with yellow phosphor material [4]. However, the thermal degradation of the phosphor can alter the color temperature, impacting the light output and color stability, and some of the blue light energy may be converted to heat. In contrast, red, green, and blue (RGB) LED combinations create white light through mixing but require complex control systems that escalate costs and system complexity [5]. One solution to streamline this process is to replace RGB LEDs with vertically stacked InGaN blue and yellow mini-LEDs, simplifying control complexity and efficiently producing white light.

Vertical stacking integration technology (VSIT) is a cutting-edge innovation gaining momentum in semiconductor and electronic component manufacturing. This technology, which utilizes photolithography alignment, mirrors the 3D packaging concepts employed in metal oxide semiconductors (CMOSs). It allows for the integration of several different functional layers, enhancing device functionality density, reducing size, and boosting overall performance. VSIT is now widely applied in three-dimensional integrated circuits (3D ICs), high-density memory storage, and optical and optoelectronic components, among other advanced technologies [6,7]. One specific application of VSIT in LEDs involves the vertical stacking of LED structures. This approach contrasts with traditional planar LED configurations and aims to deliver higher definition, lower power consumption, and superior overall performance. The vertical arrangement allows for greater pixels per inch (PPI) within a more compact area, typically providing higher brightness and potentially more energy-efficient performance in certain applications. In addition, the vertical design facilitates better heat dispersion, thus minimizing heat-related issues and damage. At present, vertical stacking integration is considered the most promising method for achieving multi-color emissions. Many research teams are exploring this technology, particularly focusing on developing high-PPI, full-color displays. There are two principal techniques for vertically stacking LEDs. The first involves integrating RGB wafers using a tunnel junction (TJ) as the connecting surface to develop monolithic stacked RGB micro-LED arrays [8,9]. This method leverages the piezoelectric field in the junction region to decrease TJ resistance in wide-gap semiconductors, enhancing current spreading and improving ohmic contacts, making TJ-connected multicolor LED stacks highly appealing [10]. However, the TJ process faces challenges, primarily because most emitters are grown through metal–organic chemical vapor deposition (MOCVD), complicating the activation of the p-type in the presence of a TJ. In addition, issues persist in boosting luminous intensity, aligning emission wavelengths, and lowering operating voltages [9]. Another approach is the vertical lamination of RGB LEDs using a polymer as an adhesive layer, simplifying the stacking process if suitable transfer techniques are available [11,12,13]. This method significantly eases fabrication challenges and holds potential for the scalable production of monolithically stacked RGB micro-LED arrays.

**Figure 1:**
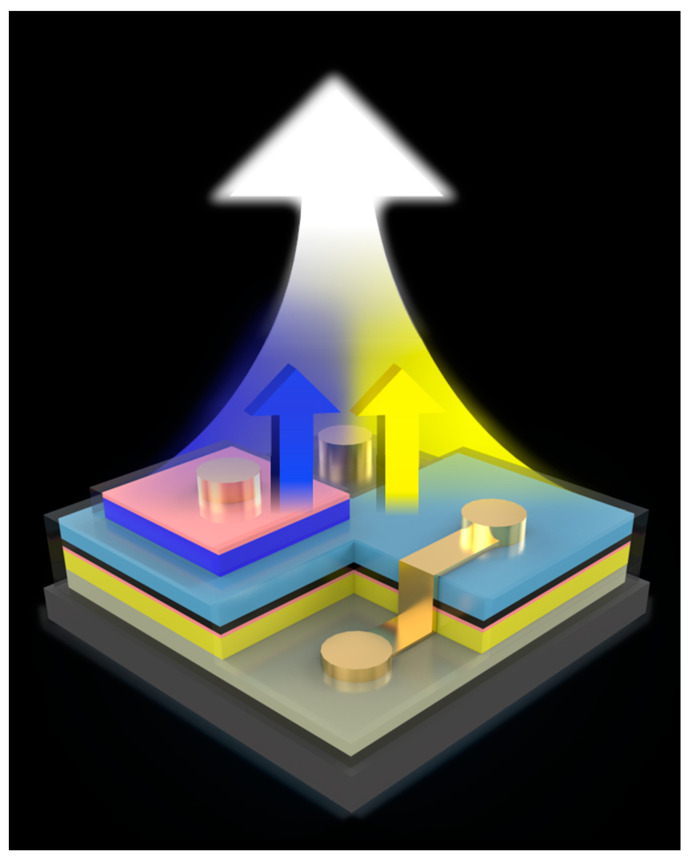
Schematic diagram of blue and yellow vertical stacked chip for light mixing.

Traditionally, most yellow LEDs have been fabricated from quaternary materials like aluminum, indium, gallium, and phosphide (AlInGaP), which exhibit instability at high temperatures and significant efficiency degradation related to size due to severe surface compounding [14,15]. Consequently, InGaN yellow LEDs, which offer stable temperature dependence and a higher light output power density (LOP), are increasingly favored for automotive LED applications. However, a key challenge with InGaN LEDs is the quantum confinement Stark effect (QCSE), a phenomenon stemming from lattice mismatches within the InGaN/GaN quantum wells (QWs) that further limits the efficiency of µ-LEDs [16,17]. To mitigate the strain on the QW, several strategies can be employed. One approach involves optimizing the substrate by inserting a stress buffer layer [18], which has shown promise in enhancing the optoelectronic properties and mitigating the QCSE [19,20,21]. Additionally, varying the thickness of the active layer [22] and employing staggered QW structures [21,23,24,25] can improve the overlap of hole and electron wavefunctions, leading to higher optical output power and electroluminescence intensity [25]. In a previous study, the introduction of a stress buffer layer significantly improved the external quantum efficiency (EQE) and mitigated QCSE phenomena in RGB LEDs. Building on these insights, this study employs a vertically stacked approach to integrate mini-LED arrays with InGaN yellow and blue epitaxial layers, incorporating stress buffer layers, as shown in Figure 1. This configuration facilitates the implementation of variable dimming effects by manipulating the electrodes on the component surfaces under different operating currents, broadening the potential applications. What is more, the temperature effects pertinent to automotive lighting have been thoroughly analyzed. The integrated devices developed in this study demonstrate considerable potential for multifunctional applications, including color-dimmable micro-light sources, automotive lighting, and outdoor signage. These advancements underscore the versatility and effectiveness of InGaN-based LEDs in overcoming traditional material limitations and enhancing LED performance in diverse settings.

## 2. Experiment and Fabrication Process

**Figure 2:**
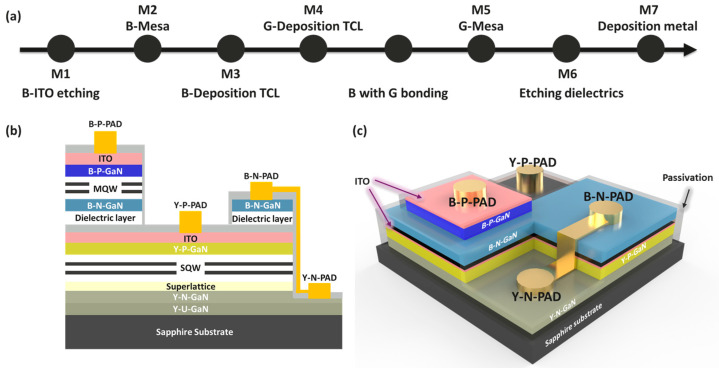
Blue and yellow vertically stacked mini-LEDs: (**a**) device fabrication flow; (**b**) schematic diagram of the cross-section; (**c**) schematic diagram of the 3D chip.

In this study, we successfully developed an independently tunable vertically stacked blue–yellow mini-LED array. Initially, we fabricated 55 × 55 μm blue mini-LEDs and 150 × 150 μm yellow mini-LEDs, then mounted the blue mini-LEDs onto the surface of the yellow mini-LEDs, constructing both blue and yellow electrodes and co-electrodes, as shown in Figure 2. The epitaxial structures of the blue and yellow μ-LEDs were grown by MOCVD, consisting of an undoped GaN buffer layer and n-GaN on a sapphire substrate. The process for the blue epitaxial structure is based on a previously developed architecture using semi-polarized blue mini-LEDs. The yellow epitaxial structure is based on another previously developed structure [17], incorporating a 30-cycle GaN (6 nm)/In0.08Ga0.92N (2.5 nm) superlattice (SL) stress release layer to minimize the effects of the QCSE and enhance the crystal quality of the subsequent InGaN QWs. In concrete terms, a hybrid QW structure comprising a low-indium blue InGaN single quantum well (SQW) and a high-indium InGaN SQW was developed for the QWs. Additionally, a superlattice electron-blocking layer (p-Al0.15Ga0.85N) and a hole-injection layer (Mg-doped p-GaN) were deposited. The wafer was then annealed in nitrogen at 1050 °C for 5 min to complete the yellow epitaxial wafer.

Next, we will introduce the vertical stacking process of the blue and yellow epitaxial wafers. Figure 2a illustrates the production process of the vertically stacked blue and yellow mini-LEDs. Blue process LEDs are symbolized by B, and yellow process LEDs are symbolized by Y. The first part involves the blue mini-LED, of which the p-GaN is composed of an InGaN/GaN SQW with a peak emission wavelength of 457 nm. The p-type GaN layer is initially coated with an indium tin oxide (ITO) layer, followed by inductively coupled plasma (ICP) dry etching to etch the n-GaN using the ICP countertop method, and several rows are defined and isolated (M1). ITO is then redeposited on the epitaxial wafer surface, and a mask and ICP are used to define the countertop structure of each blue LED pixel (M2). An ITO layer is subsequently deposited on top of the p-type GaN layer to serve as a transparent conductive layer (TCL) (M3). The sapphire substrate is then removed by laser. The process then proceeds to the yellow mini-LED section. The yellow light epitaxial wafer repeats the process of M1, with ITO deposited on the surface of the epitaxial wafer as TCL (M4). To prevent leakage currents caused by sidewall defects, a passivation layer is created using atomic layer deposition (ALD), and Al_2_O_3_ is deposited on the surface of the yellow LED epitaxial wafer. The blue epitaxial wafer is then bonded to the yellow epitaxial wafer using silicone as the adhesive. A mask and ICP are then used to define the countertop structure of the yellow LED pixel (M5), and the Al_2_O_3_ passivation layer is also applied to the vertically stacked structure using the ALD technique, followed by ICP etching of the dielectric (M6). A p-type metal is then deposited on the TCL for both blue and yellow light, and finally, an n-type metal is deposited on the n-type metal for the two light colors to create a co-electrode (M7), completing the fabrication of the mini-LED arrays. The cross-section and 3D schematic of the structure are shown in Figure 2b,c.

## 3. Result and Discussion

**Figure 3:**
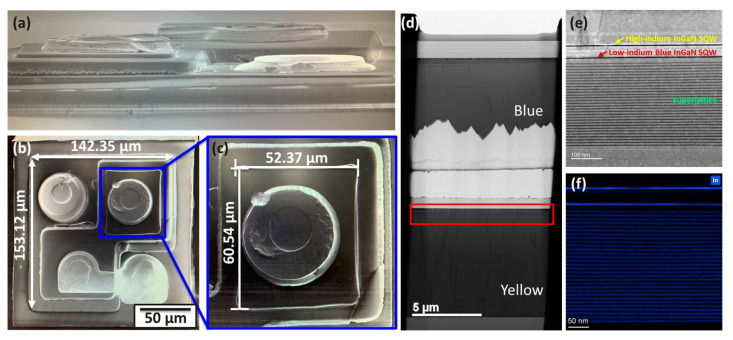
Scanning electron microscope (SEM) of blue and yellow light vertically stacked structures: (**a**) cross-section, (**b**) top view, (**c**) magnified image of blue light mini-LEDs. TEM (**d**) cross-section, (**e**) enlarged cross-section of yellow light InGaN epitaxial structure around the QW region. (**f**) EDS mapping of the In element of the yellow light InGaN epitaxial.

Figure 3a depicts the cross-section of the vertically stacked blue and yellow structures. The yellow epitaxial layer measures 153.12 μm × 142.35 μm, with the blue epitaxial layer stacked vertically on top using silicone, measuring 60.54 μm × 52.37 μm, as shown in Figure 3b–d. Currently, the primary challenge for yellow InGaN-based LEDs lies in growing InGaN QWs with low strain and high quality. This challenge stems from the high In content of long-wavelength QWs and the significant lattice mismatch with GaN as the barrier layer. The heteroepitaxial process induces higher compressive stresses and corresponding elongation in the longitudinal direction. Increasing the lattice match between the well and barrier layers could effectively enhance luminous efficiency. Therefore, in this study’s epitaxial design, the SL structure serves as a stress relief layer to mitigate the lattice compression stress of yellow light. Moreover, the low efficiency of the high In content InGaN-based LEDs primarily results from the low-temperature growth of InGaN [26,27]. To incorporate more In in the active region, a lower growth temperature is necessary, leading to increased impurity doping, surface roughness, and defect density, consequently augmenting the SRH non-radiative recombination [28]. Additionally, lattice-mismatch-associated stresses generate various defects that degrade crystallization quality. Thus, the main challenge in achieving high-efficiency InGaN-based yellow LEDs is overcoming fundamental material issues. To enhance crystal quality, the active zone was adjusted from multiple quantum wells (MQWs) to SQW, reducing defects arising during lattice relaxation and enhancing crystal quality. Furthermore, to address high In-content stress in the long-wavelength band, a hybrid quantum well structure was adopted [29]. This structure incorporates a blue SQW with a lower In content below the main InGaN yellow SQW, serving as stress release, and prevents carrier recombination in the blue SQW. An n-AlGaN potential energy barrier between the blue and yellow QWs blocks electric hole injection into the blue QW. The AlN cover layer in the epitaxial structure prevents platinum evaporation during high-temperature potential energy barrier growth. Additionally, AlN and AlGaN potential energy barriers provide tensile strains to compensate for compressive strains in the InGaN QWs [29,30], with functions in bandwidth engineering and strain compensation. The combination of AlN and AlGaN enhances optical output power. Figure 3d–f display transmission microscopy (TEM) analysis and X-ray energy dispersion analysis (EDX) of yellow epitaxial wafers. TEM observed the microstructure of the yellow epitaxial layer, cross-section dark-field STEM images were captured, and EDX mapping semi-quantitatively scanned the In element distribution.

**Figure 4:**
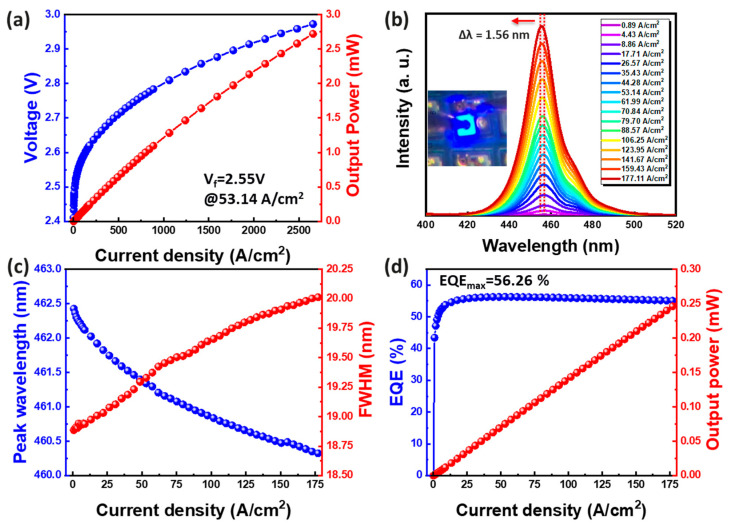
Blue mini-LED. (**a**) Voltage and output power as a function of current density. (**b**) EL spectra at different current densities. (**c**) Wavelength shift and FWHM difference as current density curves. (**d**) EQE–current density curve.

Before vertically stacking the blue and yellow lights, their optoelectronic characteristics were measured separately. Figure 4a presents the voltage (V)–current (I)–output power (L) curve of the blue mini-LED (measured with an Maya 2000 Pro, Ocean Optics, Shanghai, China). At a current density of 53.14 A/cm^2^, the forward bias voltage was 2.55 V. Subsequently, spectra were collected using a surface-coated integrating sphere system (SLM-12 system, Isuzu Optical, Hsinchu, Taiwan). Electrical signals were applied to the device via a power supply (Keithley 2400, Keithley, Hsinchu, Taiwan) and detected using a spectrometer (QE65000, Ocean Optics, Shanghai, China). Figure 4b,c displays the electroluminescence (EL) emission spectra of the blue mini-LEDs at different current densities (ranging from 0.89 to 177.11 A/cm^2^), alongside the relationship between wavelength shift and full-width at half-maximum (FWHM) variation. The blue emission wavelength shifted from 462.43 nm to 460.32 nm, with a mere 1.56 nm displacement, and the FWHM displacement was approximately 1.12 nm. Figure 4d illustrates the EQE of the blue mini-LEDs relative to the injected current. The highest EQE of 56.26% was achieved at an injection current density of 53.14 A/cm^2^ [17]. As the current injection increased to 177.11 A/cm^2^, EQE gradually decreased to 55.07%, with a 2% drop. It is well known that the efficiency degradation is caused by a non-radiative carrier loss mechanism that has little effect at low currents but becomes dominant at high currents. It is therefore to be expected that the device will gradually degrade as the applied current is gradually increased to its maximum value [17]. These results highlight the exceptional optoelectronic characteristics of blue mini-LEDs.

**Figure 5:**
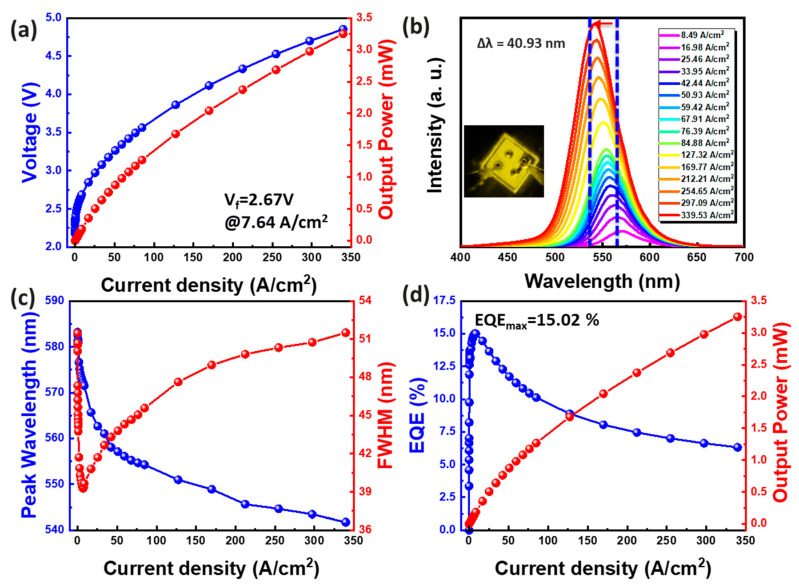
Yellow mini-LED. (**a**) Voltage and output power as a function of current density. (**b**) EL spectra at different current densities. (**c**) Wavelength shift and FWHM difference as current density curves. (**d**) EQE–current density curve.

Significantly, Figure 5a displays the V–I–L curve of the yellow mini-LED. The forward bias was 2.67 V at a current density of 7.64 A/cm^2^. Figure 5b,c demonstrates the blue-shift of emitted wavelength from 582.66 nm to 541.73 nm, with a 40.93 nm displacement, and a FWHM shift of approximately 12.2 nm across various current densities (ranging from 8.49 to 339.53 A/cm^2^). This substantial blue shift results from the shielding of the internal electric field in the c-plane InGaN QW as injection current density increases. Additionally, concerning EQE, the highest EQE of 15.02% was achieved at an injection current density of 7.64 A/cm^2^. This high EQE primarily results from increased optical power due to the presence of SL, alleviating lattice compression stress effects and enhancing epitaxial quality, consequently improving efficiency.

**Figure 6:**
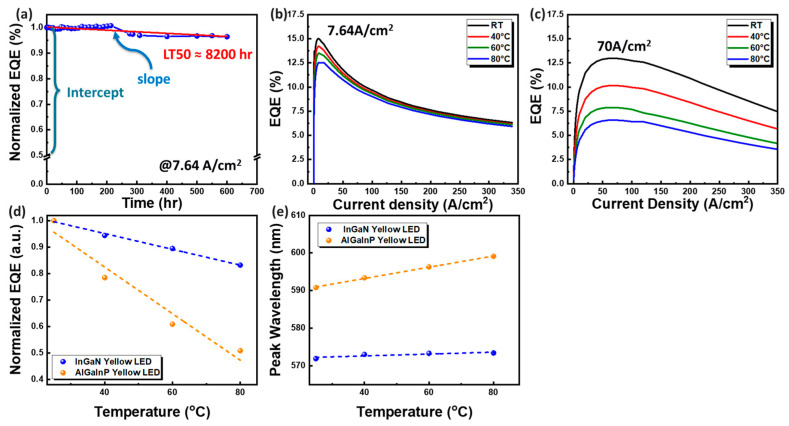
(**a**) Reliability test decay trend of InGaN yellow LEDs. (**b**) EQE trend of InGaN yellow LEDs for variable temperature measurement. (**c**) EQE trend of AlGaInP yellow LEDs. Comparison of the temperature dependence of InGaN yellow LEDs and AlGaInP yellow LEDs. (**d**) EQE. (**e**) Wavelength shift.

To meet the rigorous demands of automotive lighting applications, we conducted investigations into reliability, including the long-term aging effects and impact of different temperatures on EQE. Figure 6a illustrates the long-term aging measurement of InGaN yellow mini-LEDs. The measurement environment maintained a humidity of 60%, a temperature of 25 °C, and an operating current density set to the maximum EQE value (7.64 A/cm^2^). Following 600 h of continuous illumination, the current density only decreased by 0.036, indicating a high level of stability. To accurately gauge the device lifetime over an extended period, we can use linear extrapolation from the last 600 h of data to determine LT_50_, defined as the time it takes for the device to reach 50% of its initial value. This involves calculating 50% of the initial peak intensity using the mathematical equation

(1) $${LT}_{50}=\frac{\left(50-intercept\right)}{slope}$$

Here, the slope and intercept represent linear fitting parameters obtained from continuous aging data acquired through our equipment. Based on this calculation, the LT_50_ of the InGaN yellow mini-LEDs is predicted to be approximately 8200 h.

To ascertain the competitive advantage of InGaN yellow LEDs over AlGaInP yellow LEDs in market applications, we conducted a comparison of their EQE performance at different temperatures, as shown in Figure 6b–e. At room temperature (25 °C), the peak EQE values for InGaN yellow LEDs and commercially available AlGaInP yellow LEDs were observed at current densities of 7.64 A/cm^2^ and 70 A/cm^2^, respectively. Keeping the operating current densities fixed, we placed the devices on a heating plate to vary the measurement temperature (ranging from 25 to 80 °C). The EQE of AlGaInP LEDs decreased by 50% due to carrier overflow resulting from a small energy band shift between the active and cladding layers. In contrast, InGaN LEDs exhibited lower temperature dependence. Across the temperature range of 25 to 80 °C, the EQE of yellow InGaN LEDs decreased by only 17% due to reduced carrier spillage stemming from a larger energy band shift between the active and cladding layers, as shown in Figure 6d [31,32,33]. Additionally, yellow InGaN LEDs demonstrated superior performance in terms of wavelength shift. These findings confirm that InGaN yellow LEDs exhibit lower temperature dependence compared to AlGaInP yellow LEDs, making them more suitable for applications with higher operating temperatures.

**Figure 7:**
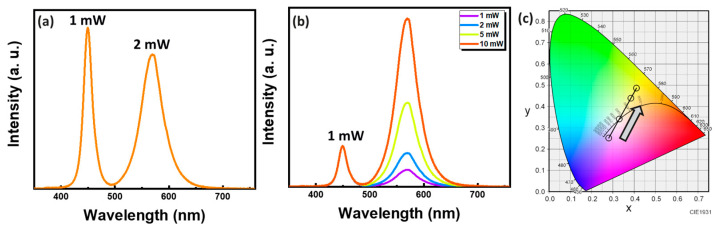
(**a**) Spectral plot of blue and yellow vertically stacked mini-LED mixing. (**b**) Trend spectra of fixed blue mini-LED operating power and varying yellow operating power (1–10 mW). (**c**) CIE 1931 chromaticity coordinate.

The vertically stacked blue and yellow mini-LEDs we designed feature separate blue and green electrodes, enabling individual control by supplying varying operating currents to achieve the modulation of the light source’s wavelength, as depicted in Figure 7. We kept the blue mini-LED operating power fixed at 1 mW while varying the yellow operating power from 1 to 10 mW. When the yellow mini-LED power is increased to 2 mW, the CIE coordinate is (0.33, 0.34), which closely approaches positive white light. The results for fixing the blue power to 1 mW and varying the yellow power (1–10 mW) are shown in Table 1. With a gradual increase in yellow mini-LED power, the light color transitions towards warm white, as illustrated in Figure 7b,c. The development of our blue and yellow vertically stacked mini-LEDs allows for adjusting the CIE coordinates by independently controlling the currents of the blue and green lights, facilitating a broader range of applications.

However, the shortcoming is that the result is a relatively narrow band in two wavelength ranges, resulting in poor color rendering, and there is still a gap in realizing automotive lighting or outdoor lighting. Therefore, we propose several solutions here. The first one is to add a layer of red quantum dots or fluorescent powder coating on the vertical stacking structure to supplement the bandwidth shortage problem. The second is to use blue, yellow, and red color epitaxial chips to improve the bandwidth problem by using our proposed simple manufacturing method. This can also improve the color rendering and make it more suitable for automotive lighting and outdoor lighting.

## 4. Conclusions

This study introduces a novel method of vertical stacking to laminate InGaN yellow and blue epitaxial layers, incorporating a stress buffer layer to create mini-LED arrays. This approach enhances optoelectronic characteristics and structural stability, simplifying LED design by reducing the need for RGB configurations, thus lowering costs and system complexity. The vertically stacked design achieves high-density, efficient white light production suitable for multifunctional applications, including dimming, automotive lighting, and outdoor signage. Yellow InGaN LEDs, featuring an SL structure as a stress release layer, demonstrate outstanding optoelectronic characteristics and relieve lattice compression stress. Additionally, InGaN yellow LEDs exhibit lower temperature dependence compared to AlGaInP yellow LEDs, making them more suitable for high-temperature applications. Long-term aging measurements show that InGaN yellow mini-LEDs have high stability, with an estimated LT_50_ of 8200 h, underscoring their reliability. Experimental results indicate that stacked yellow and blue mini-LEDs exhibit exceptional performance in terms of luminous efficiency, wavelength precision, and thermal stability. The individually controllable blue and yellow vertically stacked mini-LEDs enable easy wavelength regulation through different operating currents, offering significant advantages for automotive lighting and other optoelectronic applications. The integration of the stress buffer layers effectively mitigates lattice compression stress, improving both optoelectronic properties and long-term reliability. The independently tunable vertically stacked blue-yellow mini-LED array developed in this study offers significant advancements in lighting system design and application, with potential implications for both automotive and general illumination markets. These findings highlight the versatility and effectiveness of InGaN-based LEDs in enhancing performance and overcoming traditional material limitations. However, the current approach shows some shortcomings in terms of color rendering. We hope to continue optimizing this structure in the future to make it more suitable for applications such as automotive lighting and outdoor lighting.

## Figures and Tables

**Table 1:** Comparison of a fixed blue power of 1 mW and changing yellow power.

	1 mW	2 mW	5 mW	10 mW
CIE	(0.28, 0.25)	(0.33, 0.34)	(0.38, 0.44)	(0.41, 0.49)
CCT (K)	13891.86	5604.16	4273.78	3990.95

## Data Availability

The data presented in this study are available from the corresponding author upon reasonable request.
